# Effects of Automation on Sustainability of Immunohistochemistry Laboratory

**DOI:** 10.3390/healthcare9070866

**Published:** 2021-07-08

**Authors:** Marija Đorđević, Maja Životić, Sanja Radojević Škodrić, Jelena Nešović Ostojić, Jasmina Marković Lipkovski, Jelena Filipović, Sanja Ćirović, Sanjin Kovačević, Duško Dunđerović

**Affiliations:** 1 Faculty of Organization Sciences, University of Belgrade, 11010 Belgrade, Serbia; 2 Institute of Pathology, Faculty of Medicine, University of Belgrade, 11000 Belgrade, Serbia; majajoker@gmail.com (M.Ž.); sanjaskodric@gmail.com (S.R.Š.); acal@matf.bg.ac.rs (J.M.L.); jecapeleca@gmail.com (J.F.); sanjactlll@gmail.com (S.Ć.); 3 Republic Fund of Health Insurance, 11000 Belgrade, Serbia; 4 Department of Pathophysiology, Faculty of Medicine, University of Belgrade, 11000 Belgrade, Serbia; jelena.nesovic-ostojic@med.bg.ac.rs (J.N.O.); sanjin.kovacevic@med.bg.ac.rs (S.K.)

**Keywords:** automated immunostainer, automation, cost reduction, immunohistochemistry

## Abstract

The COVID-19 pandemic that hit the world recently caused numerous changes affecting the health system in every department. Reduced staff numbers, mostly due to illness, led to an increase in automation at every stage of laboratory work. The immunohistochemistry (IHC) laboratory conducts a high volume of slide staining every day. Therefore, we analyzed time and total costs required to obtain IHC slides in both the manual and automated way, comparing their efficiency by processing the same sample volume (48 microscope slides—the maximum capacity that an automated immunostainer—DAKO, Autostainer Link 48, Part No AS48030—can process over a single cycle). The total IHC procedure time to run 48 slides manually by one technician was 460 min, while the automated process finished a cycle within 390 min (15.22% less time). The final cost of a single manual IHC slide was 12.26 EUR and 7.69 EUR for slides labeled in the automated immunostainer, which reduced final costs by 37.27%. Thus, automation of the IHC procedure reduces the time and costs of the IHC process, contributing significantly to the sustainability of the healthcare system during the COVID-19 pandemic, overcoming insufficient human resources.

## 1. Introduction

A broad spectrum of molecular testing in medicine influences patients’ diagnoses, treatment, and/or prognoses. Thus, physicians and other medical staff need to balance these requirements and cost procedures that influence the budget of medical institutions and insurance companies. Pathologists use a wide spectrum of analysis daily. However, immunohistochemistry is the routine that is performed at a high volume every day. Thus, seeking new approaches to increase the volume of IHC stained slides while decreasing the laboratory costs and time spent on manual application of IHC protocols led pathologists to introduce the automated process into their laboratories [1,2]. However, despite numerous advantages of automated IHC labeling, which allows better standardization [3,4,5], reliability, and reproducibility of IHC, particularly in the clinical setting [6], the introduction of an innovative automated immunostaining has never undergone profound analysis of its efficacy, and only suggestions and speculations of costs and time reductions exist [7,8,9,10,11]. Automation improves performances in clinical laboratories [12], but it still represents a great challenge, considering the implementation of numerous changes required for the successful automation of procedures, involving a completely new approach to managing the existing processes. Moreover, it is very important to choose carefully the type of innovative equipment that will automate the process, either completely or partially [13,14]. IHC staining cannot be completely automated, since there are several steps in the IHC protocol that depend on manual work [15]. This process is considered the process of partial automation, whereas a fully automated process would be conducted completely by machines, including all steps during biopsy sample preparation for further IHC procedure. These steps are acquisition of formalin-embedded paraffin-fixed tissue blocks, their cutting using microtome, slide labeling, and IHC staining. Finally, fully automated procedures would also require quality control as well as slide delivery and interpretation [7]. However, regardless of these disadvantages, partial automation is extremely important because it allows laboratory technicians to be focused precisely on specific activities that require their expertise, skills, and concentration, while routine activities could be performed by machines through the automation process [16,17]. In addition to the aforementioned economic benefits, the optimization of the process achieved by automation has an exceptional significance considering turnover rate and shorter time to obtain the histopathological results, allowing physicians to get precise diagnoses and act in a timely manner by prescribing the appropriate treatment [18]. Numerous laboratories for clinical microbiology that automated some or most of their processes have found that testing can be done accurately, while reducing the time required for analyses, improving laboratory efficiency, and increasing flexibility in terms of skill levels required to perform laboratory work [1,19,20,21]. Furthermore, new technologies create the ability to perform steps that could not be performed by manual work for any reason. Automation of processes enables reduction of human mistakes, allowing successful processing independent (or dependent to a lesser extent) on human knowledge, skills, and expertise [22,23]. Finally, both patients and institutions would achieve great benefits. Patients would get quick and accurate results, while institutions would have higher turnover rates and be able to create new jobs.

Decisions about the implementation of automated immunostainers should be based on the performance of machines, the duration of a single staining cycle, the cost of the machine and its annual service and depreciation, the quality of IHC labeled slides, etc. Furthermore, this decision may also depend on requests of departments, the length of technicians’ workday, and their salaries.

The automation of IHC procedures was introduced in our lab a few years ago. The adjustment of new approaches to routine work performed by technicians and their adaptation to new methods, including avoiding automation due to widely used established habits, have led our laboratory team to perform most of the analyses by the manual method. However, the COVID-19 pandemic caused a number of changes. The key question became how to ensure sustainability and stability in the continued work of the laboratory with limited and reduced resources. Considering that the healthcare system was extremely affected in every segment, in addition to the available resources in terms of professional staff, special attention is paid to the financial effects. The COVID-19 pandemic caused a decrease in the capacity of the laboratory considering professional staff, due to their redistribution to other places within our healthcare system; reduced staff due to illness and prolonged absences from work; as well as increased analyses due to reduced capacity in related laboratories. All these factors required increased automation throughout the entire process of laboratory work.

Since the automated immunostainer is used at full capacity, the aim of this research is to calculate the effects of automation on laboratory resources. Considering that IHC is widely used in our lab and appreciating an increased effort to create a modern and effective healthcare system have led us to calculate the efficacy of the automation process in the immunohistochemistry laboratory, comparing it with standard manual protocols at the Institute of Pathology, Faculty of Medicine, University of Belgrade.

## 2. Materials and Methods

### 2.1. Biopsy Specimens

Human biopsy samples, routinely formalin-fixed and paraffin-embedded (FFPE) tissues, which were processed by the Immunohistochemistry Laboratory of the Institute of Pathology, Faculty of Medicine, University of Belgrade for IHC staining procedure, were used in this study. The specimens included a wide range of sample types including malignant and non-malignant tissues that were processed with various IHC staining procedures using laboratory-validated methods, following manufacturer-recommended protocols.

### 2.2. Protocols for Immunohistochemistry

The manual IHC staining procedure and IHC automation process in an automated immunostainer with all steps and their durations, as well as with required reagents, are illustrated in Table 1. The automated immunostainer used in this study works at a full capacity of 48 slides per one IHC cycle. Thus, in order to precisely compare time and costs per microscope slide, experimentally in the current research, a technician performed the IHC protocol using the same amount as the automated immunostainer (48 slides). For this experiment, the technician used the same tissue samples and the same antibodies in both procedures, in order to make direct comparisons. Slide labeling, FFPE tissue cutting, and slide drying are the first three steps both in manual and automated protocols, which are performed manually. Moreover, the last 10 steps in both procedures are also performed manually.

### 2.3. Reagents and Instrumentation

Reagents used to perform the IHC analyses were obtained from various manufacturers, mainly from Sani-Hem (Novi Bečej, Serbia), DAKO (Agilent, Santa Clara, CA, USA), Thermo Fisher Scientific (Waltham, MA, USA), Abcam (Cambridge, UK), and Molar Chemicals Kft (Halásztelek, Hungary). An automated immunostainer (DAKO, Autostainer Link 48, Part No AS48030, Agilent, Santa Clara, CA, USA) was used in the current investigation. A detailed description of the automated procedure protocols is beyond the scope of the current work.

Reagent costs were calculated from the volumes and amounts of reagents required to process one IHC specimen manually (Table 2) or in the automated immunostainer processor (Table 3).

Superfrost microscope slides (Thermo Fisher Scientific) were the same in both manual and automated procedures. The cost of a single microscope slide was obtained by dividing the price of a pack (23.00 EUR) by the number of units within the manufacturer’s pack (72 pieces). Therefore, the cost of this item per single IHC run was 0.32 EUR.

Xylene, as well as alcohol (100%, 96% and 70%), are used in the cuvette capacity of 100 mL, containing 16 slits for slides. A single cuvette filling with the aforementioned solutions could be used at least 4 times and afterwards discharged. This means that a manufacturer’s pack of 1 L could provide 10 cuvette fillings, providing 640 IHC slide preparations (10 × 16 × 4). The costs of xylene and alcohol are almost negligible.

The manual IHC procedure requires different heat-induced epitope retrieval (HIER) reagents. Depending on the antibody, the following HIER reagents were used: Citrate buffer (pH 6.0), Tris-EDTA buffer (pH 8.0), and Tris-EDTA buffer (pH 9.0). The prices of a 1000 mL manufacturer’s pack of reagents were, respectively, 103.33 EUR, 250.00 EUR and 95.00 EUR. An average price of these reagents was used as the price of HIER antigen retrieval reagent presented in Table 2. The sum of 448.33 EUR was divided by 3, and 149.44 EUR was used for further calculations. HIER is used in a volume of 250 mL per cuvette with a rack containing 20 microscope slides. Thus, a 1000 mL manufacturer’s pack is suitable for 80 microscope slides. The cost of HIER reagent per slide is 1.87 EUR.

The PBS washing buffer (10×) of 1000 mL manufacturer’s volume was diluted 10 times and then used in the cuvette of 100 mL volume containing 16 slits for slides. Therefore, the manufacturer’s pack could wash 1600 slides. As such, the price of a single IHC slide washing was 0.006 EUR. However, the PBS washing step is applied 11 times per single IHC cycle, contributing to an increase in PBS reagent cost by 0.06 per slide in the entire manual IHC protocol.

In the manual protocols, different antibodies were used, and the price presented in the cost analysis was calculated as an average price. The antibody price for IHC in the automation procedure was fixed. The amount of antibody used differs between manual and automated protocols; thus, the cost of antibodies was factored into the costs here. An automated procedure uses 150 µL of ready-to-use (RTU) antibody per single IHC slide. A manual IHC procedure uses different antibody dilutions. However, for the current study, 1:100 dilutions are used. This means that 100 µL of diluent was mixed with 1 µL of antibody in the manual IHC protocol, per single slide.

The kit for detection of antibody binding used in the manual IHC procedure could provide 833 IHC slide visualizations, because 150 µL was used per slide, and the manufacturer’s pack provides 125 mL volume. In the automated process, the kit is composed of 600 tests, and the same amount of slides could be visualized.

The quantity of 3,3′-diaminobenzidine (DAB) used in a manual IHC run was 300 µL. Therefore, the manufacturer’s pack could provide 416 slides, as illustrated in Table 2.

Hematoxylin was used in both manual and automated IHC staining in the same way, with a cuvette capacity of 100 mL containing 16 slits for slides. A single cuvette filling with the aforementioned solution could be used at least 4 times and afterwards discharged. This means that a manufacturer’s pack of 1 L could provide 10 cuvette fillings, providing 640 IHC slides preparations (10 × 16 × 4). These costs are almost negligible.

Canada balsam was provided by the manufacturer in a 100 mL volume. Considering usage of 40 µL per IHC slide, 2500 slides could be covered by a single pack, as shown in Table 2 and Table 3.

Coverslip costs are the same in both processes, as shown in Table 2 and Table 3.

## 3. Results

At the beginning of this section, we are going to present comparative analyses of manual and automated IHC procedures by implementation of the same sample volumes, in order to precisely calculate the price of a single IHC slide and to assess the time required to finalize the entire IHC cycle.

### 3.1. IHC Cycle Duration

In the present study, we analyzed the duration of a single IHC cycle performed manually and in an automated immunostainer using the same microscope slide volumes in both procedures (48 slides), as illustrated in Table 1. For the current experimental design, both manual and automated runs were performed with four different antibodies, in order to make direct comparisons of two procedures accurate as far as possible. In real daily situations in the lab, an automated IHC single cycle is usually performed with the most frequently used antibodies by our pathologists, and an automated immunostainer mostly works with four to six different antibodies. On the other hand, real manual run IHC protocols involve many different antibodies performed by one technician, thus slightly increasing the time required for reagent preparation, such as dilution of numerous antibodies. The total procedure time in the automated process for full capacity working (48 slides) is fixed at the maximum volume, and it slightly depends on the hands-on time during the preparation steps and at the finalization of the staining procedure (Table 1). The total IHC procedure time to run 48 slides manually by one technician was 460 min (7 h 40 min), while the automated process finished a cycle within 390 min (6 h 30 min), resulting in 15.22% less time to finish the cycle. Slide drying is considered to be an almost passive process. However, it increases the total time required to obtain slides for further processing according to IHC protocol.

### 3.2. Cost Analysis

The total cost of a single IHC slide represents the sum of different costs. Both in manual and automated procedures, the total cost includes cost of reagents used for immunostaining, cost of technicians’ work, consumables, and resources. In addition to these costs, an automated cost considers depreciation of the automated immunostainer, as well as costs of an annual automated immunostainer service.

#### 3.2.1. Cost of Reagents Used to Run IHC

All reagents required for the manual and automated IHC staining procedure are listed in Table 2 and Table 3. The cost of a single reagent per one microscope IHC slide was calculated based on the price of the manufacturer’s pack that was divided by the final number of units, which can be used from the pack. The costs of reagents used for a single immunohistochemistry run (one IHC microscope slide) in the manual procedure are represented in Table 2, while the costs of reagents in the automated procedure are listed in Table 3. These tables include information with regard to price and quantity, which are calculated per slide.

It was found that the price of IHC reagents per slide was significantly lower in the automated process (7.09 EUR) than the price calculated for the manual protocol (11.63 EUR).

Costs for hands-on time were calculated assuming a standard Serbian laboratory personnel (technician) cost of 3.83 EUR per hour (an average monthly gross salary for a laboratory technician in Serbia is 80,983.00 RSD [24], i.e., 674.86 EUR at the euro exchange rate of 120.00 RSD; salary per hour was calculated for 22 business days per month and 8 working hours per day) multiplied by the actual time the technician was manually handling the specimens (48 specimens, manually or in the automated immunostainer). Times for specimen analysis and interpretation were not considered in this study. Since the automated immunostainer can process up to 48 slides, the time and number of slides in the manual run were also measured for the same capacity in order to allow direct comparison to the automated method.

Costs for hands-on time for the processes during the automated method were calculated for 80 min of technician work, while the whole manual run took 400 min of technician work. Since one minute of technician work costs 0.06 EUR, the final cost of technician’s work per IHC cycle (48 IHC microscope slides) was 24.00 EUR in the manual IHC labeling and 4.8 EUR in the automated procedure. Thus, the cost of technicians’ work per single IHC slide was 0.50 EUR in the manual run and 0.10 EUR in the automated way (Table 4).

#### 3.2.2. Cost of Consumables

Consumables costs include microtubes, pipette tips and nitrile gloves, and they are the same in both procedures. Table 4 includes information with regard to the price and quantity calculated per slide. The price of IHC consumables per slide was 0.12 EUR.

#### 3.2.3. Cost of Resources

During work in the laboratory, some other resources should also be considered, such as electricity and water supply. The current cost analysis included the cost of electricity, while water supply cost could not be analyzed due to the inability to separate lab water consumption from other spaces within the building, as well as due to the inability to separate consumption for immunohistochemistry procedure from other staining procedures performed in the same lab. Electricity costs were calculated according to the available information on power consumption costs [25], using manufacturer information for specific equipment and devices consumption used both in manual and automated IHC procedures. The following parameters were used for the electricity cost analysis: the billing period of 30 days, single-rate meter, three-phase connection type, approved power of 6.90 kW, and no public service fee. Resources (electricity) for the manual and automated IHC staining procedure are listed in Table 4, which also includes information with regard to price and quantity calculated per slide. The electricity price per slide was 0.01 EUR for both processes.

#### 3.2.4. Cost of Depreciation of Automated Immunostainer

The total depreciation cost was calculated for the automated immunostainer and connected computer together, based on the price of 35,000 EUR (paid for both devices together) and planned usage time of 10 years (with an average 250 working days per year). Thus, depreciation cost was considered only in automated procedure and was 0.29 EUR per slide, as shown in Table 4.

#### 3.2.5. Costs of Annual Automated Immunostainer Service

An annual automated immunostainer service is mandatory according to manufacturer’s instructions, in order to maintain the accurate and valid staining procedure. Thus, the total cost of the automated IHC procedure also takes into account the automated immunostainer annual maintenance cost of 1000.00 EUR. However, this is almost negligible considering the high annual volume of IHC slides performed in an automated immunostainer (12,000 slides). It is important to mention that due to the complete cost-effectiveness of the use of the equipment, the automated immunostainer never turns on unless there is a sufficient volume of microscope slides. So, the automated immunostainer always works at full capacity. The cost of an annual automated immunostainer service was 0.08 EUR per slide.

#### 3.2.6. Final Cost of Single Immunohistochemistry (IHC) Microscope Slide

Table 4 describes in detail all the variables that influence the final cost of an IHC slide. Thus, the prices per IHC slide were 12.26 EUR for manually processed slides and 7.69 EUR for slides labeled in the automated immunostainer.

### 3.3. Models of Routine Work in the Immunohistochemistry Laboratory before and during the COVID-19 Pandemic

**Figure 1:**
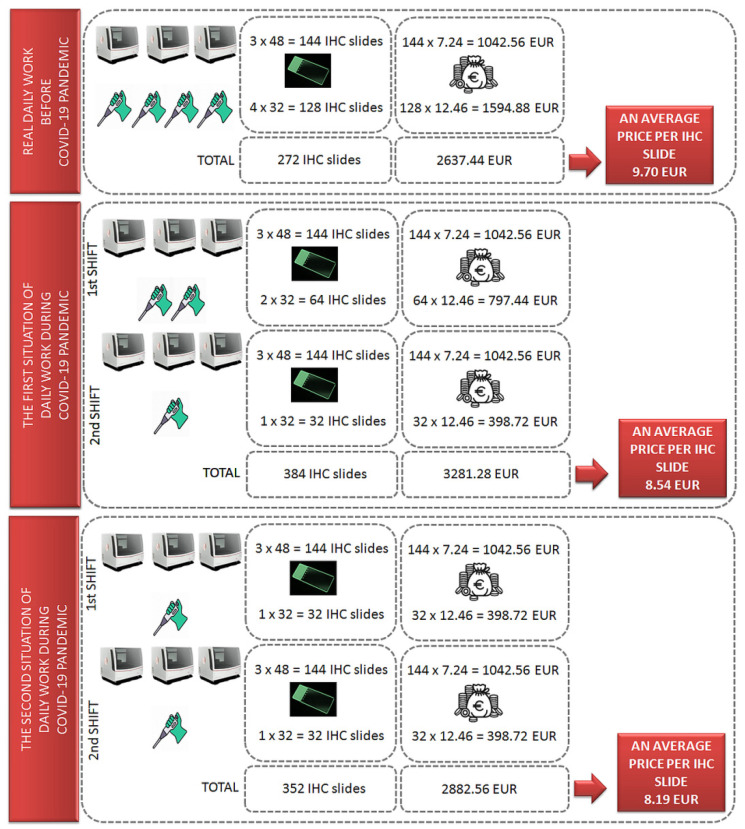
Models of routine work in the immunohistochemistry laboratory before and during the COVID-19 pandemic.

Figure 1 describes in detail all variables that influence the final average cost of an IHC slide, depending on the shifts of laboratory technicians and on the number of automated immunostainers, as well as on the number of their cycles per day. Our lab usually receives between 250 and 400 IHC samples. Thus, in order to provide IHC results without delay, we organized shift work in the lab during the COVID-19 pandemic, and we noticed that IHC staining at higher volume in the automated immunostainer reduced the average costs of a single IHC slide, as illustrated in Figure 1.

## 4. Discussion

The sustainability of the healthcare system relies heavily on careful management of health services, balancing medical, economic, and social aspects. Thus, precise analysis and measurements of health service costs and time required to obtain final results are crucial. The current study compares the final cost of IHC slides processed manually with IHC processing in the automated immunostainer, considering also the time required for one IHC cycle analysis.

Interestingly, the automated instrumentation, which allows simultaneous processing of up to 48 specimens at one time, facilitates significant cost saving, contributing to the reduction of final costs by 37.27% compared to the manual procedure. Although it has been previously suggested by other researchers that automation could reduce total costs of IHC [7,8,9,10,11], for the first time here, we performed profound and precise calculations involving direct comparison of automated procedure and manual process. Similarly, Zanatta et al. observed savings from 55 to 89% for an automated FISH analysis procedure compared to costs in manual procedure [26]. Regardless of the type of analysis in a molecular pathology laboratory, these results indicate significant cost savings using automated processes. However, there are still suspicions concerning the introduction of automation in IHC laboratories worldwide. Most concerns relate to staff numbers, which usually remain the same; therefore, the salary costs remain and the investment in an automated immunostainer is believed to be an additional cost, increasing the budget of the department. However, our current study clearly indicates that investment in equipment does not increase the overall budget, since long term usage, despite depreciation and annual service costs, saves a significant amount of money.

Use of the automated immunostainer allowed for hands-free, walk-away time to the order of 70 min (460 min for manual IHC staining time versus 390 min hands-on time for the automated immunostainer), which gives laboratory staff time to perform other activities and protocols, while the IHC protocol in the automated immunostainer is executed. Altogether, these results are significant, as IHC is often considered a labor-intensive methodology and therefore cost and time savings, and ease of use are high priorities for IHC users [27]. Since the modern molecular pathology laboratories are continuously requested to accommodate higher testing volumes and increasing numbers of biomarkers and technologies, through robust automation, laboratory staff can accommodate more assays, produce more reliable results, and increase hands-free time for other important tasks. Moreover, a low turnover analysis rate produced in non-automated laboratories would be improved by the implementation of automated procedures, thereby producing higher analysis volumes of higher quality, with decreased total costs and reduced technicians’ workload [27]. Considering also social aspects, patients would be more satisfied with the decreased time required to obtain a final histopathology report that usually depends on IHC analysis and which significantly affects further therapy options and disease outcomes.

The level of cost saving experienced with the automation of IHC with the automated immunostainer is directly related to the hourly salary of a laboratory worker and the amount of hands-on time required for manual IHC processing, as well as being related to service costs and the intervals of maintenance that also vary from country to country and instrument to instrument. At our institute, there is annual preventative maintenance, which also covers unpredictable costs and services during the year. Nonetheless, the system operates robustly and thus far has not malfunctioned or required any significant maintenance. Thus, as presented here, these maintenance costs increased the price per microscope slide by only 0.08 EUR, which can almost be neglected. The current study is a specific example of when automating IHC was applied in this particular laboratory. Nonetheless, the authors believe that a similar trend would be observed for most laboratories, especially those in developed countries where salaries are higher and where equipment is even cheaper, because ordering from developing countries includes customs duties that should also be paid.

An important aspect that requires consideration when new molecular protocols are developed and/or automated is the specimen quality and assay failure rate [28]. The laboratory of our institute is a referent IHC testing hub, receiving numerous specimens from various medical centers and hospitals in the area. As such, there is little standardization of specimen preparation and storage between centers, making a direct comparison of failure rates between the manual and automated IHC protocols difficult.

Moreover, although the automated IHC is not an innovative procedure and is widely used in developed countries, developing countries are delaying the decision to introduce automated procedures, which is mostly due to the high cost of instrumentation along with the maintenance and other required modern equipment [29]. The current study, as well as numerous previous investigations, has shown that automation completely or partially eliminates the existing limitation of manually performed procedures, creating further improvements and opportunities [30]. Here, we provide the evidence that the introduction of automation in all countries can significantly contribute not only to savings within healthcare systems but can also be an important step in overcoming reduced human resources (laboratory staff) in the current COVID-19 pandemic situation.

## Figures and Tables

**Table 1:** Immunohistochemistry (IHC): protocol steps and IHC cycle duration in manual run and automated procedure using automated immunostainer (48 slides).

Protocol Steps	IHC Protocol	Duration (min)	
		Manual	Automated
1	Slide labeling	24	24
2	FFPE cutting	24	24
3	Slide drying	60	60
4	Xylene	15	125 *
5	Alcohol 100%	6	
6	Alcohol 96%	4	
7	Alcohol 70%	2	
8	Washing step—distilled water (dH_2_O)	4	
9	Cooking and cooling (HIER)	75	
10	Washing step—distilled water (dH_2_O)	5	
11	Peroxidase block—3% H_2_O_2_	10	125 *
12	Washing with PBS (2 × 5 min)	10	
13	Protein block—1% BSA	5	
14	Application of primary antibody	12	
15	Primary antibody incubation step	60	
16	Washing with PBS (3 × 5 min)	15	
17	Application of secondary antibody	8	
18	Secondary antibody incubation step	10	
19	Washing with PBS (3 × 5 min)	15	
20	Application of tertiary antibody	8	
21	Tertiary antibody incubation step	15	
22	Washing with PBS (3 × 5 min)	15	
23	DAB	18	
24	Washing step—dH_2_O	8	
25	Hematoxylin	2	2
26	Washing step—dH_2_O	2	2
27	Ammonia	1	1
28	Washing step—dH_2_O	2	2
29	I 100% alcohol	1	1
30	II 100% alcohol	1	1
31	Xylene + 100% alcohol (ana partes)	10	10
32	I Xylene	5	5
33	II Xylene	5	5
34	Canada balsam and covering	3	3
	Total time [min]	460	390

*—duration of automated steps.

**Table 2:** Costs of reagents used for single immunohistochemistry run (one IHC microscope slide) in manual procedure.

No.	Reagents	Volume of Manufacture Pack (Unit of Measure)		Price (EUR)	Quantity Used in a Single Run	Total Number of Units from Manufacture Pack	Reagent Cost of a Single IHC Slide (EUR)
1	Superfrost microscope slides	72	pcs	23.00	1 pcs	72	0.32
2	Xylene	1000	mL	2.67	100 mL	640	0.00
3	Alcohol 100%	1000	mL	3.48	100 mL	640	0.01
4	Alcohol 96%	1000	mL	2.58	100 mL	640	0.00
5	HIER antigen retrieval reagent	1000	mL	149.44	250 mL	80	1.87
6	PBS washing buffer (10×)	1000	mL	9.33	100 mL	1600	0.06
7	Primary antibody	100	µL	591.66	1 µL	100	5.92
8	Antibody diluent	125	mL	125.00	100 µL	1250	0.10
9	Kit for detection of antibody binding	125	mL	2150.00	150 µL	833	2.58
10	DAB Buffer substrate and chromogen	125	mL	294.00	300 µL	416	0.71
11	Hematoxylin	1000	mL	2.95	100 mL	640	0.00
12	Canada balsam	100	mL	46.00	40 µL	2500	0.02
13	Coverslips	100	pcs	3.85	1 pcs	100	0.04
Total							11.63

**Table 3:** Costs of reagents used for single immunohistochemistry run (one IHC microscope slide) in automated procedure.

No.	Reagents	Volume of Manufacture Pack (Unit of Measure)		Price (EUR)	Quantity Used in a Single Run	Total Number of Units from Manufacture Pack	Reagent Cost of a Single Run (EUR)
1	Superfrost microscope slides	72	pcs	23.00	1 pcs	72	0.32
2	Primary antibody—ready to use (RTU)	12	mL	250.00	150 µL	80	3.12
3	EnVision FLEX visualization system	600	tests	2150.00	1 test	600	3.58
4	Hematoxylin	1000	mL	2.95	100 mL	640	0.00
5	Alcohol 96%	1000	mL	2.58	100 mL	640	0.00
6	Alcohol 100%	1000	mL	3.48	100 mL	640	0.01
7	Xylene	1000	mL	2.67	100 mL	640	0.00
8	Canada balsam	100	mL	46.00	40 µL	2500	0.02
9	Coverslips	100	pcs	3.85	1 pcs	100	0.04
Total							7.09

**Table 4:** Variables that influence final cost (EUR) of a single immunohistochemistry (IHC) microscope slide performed in manual and automated protocols.

No	Type of Costs		Manual Protocol	Automated Protocol
1	Reagent		11.63	7.09
2	Cost of technicians’ work		0.50	0.10
	Gross technician monthly salary (EUR)		674.86	674.86
	Number of working days monthly		22	22
	Number of working hours monthly		176	176
	Price of technicians’ work per 1 h		3.83	3.83
	Price of technicians’ work per 1 min		0.06	0.06
	Total technicians’ hands-on time per single IHC cycle (min)		400	80
	Total technicians’ hands-on time per single IHC slide (min)		8.33	1.67
3	Consumables		0.12	0.12
	Microtubes 1.5 mL		0.03	0.03
	Pipette tips 200 μl		0.05	0.05
	Pipette tips 0.2–10 μL		0.03	0.03
	Nitril gloves		0.01	0.01
4	Resources (electricity)		0.01	0.01
	Monthly electricity consumption (kWh)	Illumination	61.14	8.55
		Microscope	0.26	0.40
		Automated cooking and cooling	30.25	30.25
		Automated immunostainer	/	60.50
		Fridge	63.36	63.36
	Total monthly electricity consumption (kWh)		155.00	163.06
	The price of total monthly electricity consumption (EUR)		14.44	14.91
	Number of IHC cycle per day		1	1
	Number of IHC microscope slides per cycle per day		48	48
	The price of electricity consumption calculated per single IHC slide (EUR)		0.01	0.01
5	Depreciation costs of automated immunostainer		/	0.29
	Automated immunostainer price (EUR)		/	35,000
	Expected automated immunostainer usage time (years)		/	10
	Number of working days per year		/	250
	Number of IHC slides per day		/	48
	Number of IHC slides per year		/	12,000
	Number of IHC slides within the expected usage automated immunostainer time		/	120,000
6	Annual automated immunostainer service		/	0.08
	Annual service cost (EUR)		/	1000.00
	Annual IHC slide volume (number of IHC slides)		/	12,000
	Total cost (1 + 2 + 3 + 4 + 5 + 6)		12.26	7.69
